# Calcinosis in juvenile dermatomyositis is influenced by both anti-NXP2 autoantibody status and age at disease onset

**DOI:** 10.1093/rheumatology/keu259

**Published:** 2014-07-01

**Authors:** Sarah L. Tansley, Zoe E. Betteridge, Gavin Shaddick, Harsha Gunawardena, Katie Arnold, Lucy R. Wedderburn, Neil J. McHugh

**Affiliations:** ^1^Royal National Hospital for Rheumatic Diseases NHS Foundation Trust, ^2^Department of Pharmacy and Pharmacology, University of Bath, ^3^Department of Mathematical Sciences, University of Bath, Bath, ^4^Department of Rheumatology, Southmead Hospital, North Bristol NHS Trust, Bristol, ^5^Rheumatology Unit, University College London Institute of Child Health and ^6^Arthritis Research UK Centre for Adolescent Rheumatology at University College London, University College London Hospital and Great Ormond Street Hospital, London, UK.

**Keywords:** juvenile dermatomyositis, autoantibody, calcinosis, age

## Abstract

**Objective.** Calcinosis is a major cause of morbidity in JDM and has previously been linked to anti-NXP2 autoantibodies, younger age at disease onset and more persistent disease activity. This study aimed to investigate the clinical associations of anti-NXP2 autoantibodies in patients with JDM stratified by age at disease onset.

**Methods.** A total of 285 patients with samples and clinical data were recruited via the UK Juvenile Dermatomyositis Cohort and Biomarker Study. The presence of anti-NXP2 was determined by both immunoprecipitation and ELISA. Logistic regression analysis was performed to assess the age-dependent relationship between anti-NXP2 and the development of calcinosis and disease activity measures.

**Results.** We identified anti-NXP2 autoantibodies in 56 patients (20%). While in all patients younger age at disease onset was associated with an increased risk of calcinosis and this relationship was nearly linear, anti-NXP2 autoantibodies substantially increased the risk of calcinosis across all ages (*P* = 0.025) and were detectable prior to calcinosis development. Children with anti-NXP2 autoantibodies had a greater degree of weakness (median lowest ever Childhood Myositis Assessment Score 29.6 *vs* 42) and were less likely to be in remission at 2 years post-diagnosis. No difference in disease activity was seen 4 years post-diagnosis.

**Conclusion.** Children diagnosed at a young age have a high risk of calcinosis regardless of autoantibody status. However, the presence of anti-NXP2 autoantibodies substantially increases the risk of calcinosis across all ages and is associated with disease severity.

## Introduction

Our group has previously demonstrated that antibodies to a 140 kDa protein detected by protein immunoprecipitation (known as anti-p140 or P140) in JDM were strongly associated with the development of calcinosis, a major cause of morbidity [1]. The presence of autoantibodies to a 142 kDa antigen (anti-MJ) in JDM has also been shown by others to be associated with a severe disease course, worse functional status and persistent disease activity [2]. It is now clear that the target of these autoantibodies is a 140 kDa protein, NXP2 (molecular weight 140 kDa). Anti-NXP2 autoantibodies are common in JDM and identifiable in 13–23% of patients [1, 2].

JDM is a heterogeneous disease and autoantibodies are potentially useful biomarkers to divide patients into homogeneous subgroups and inform on prognosis. Anti-NXP2 autoantibodies are of particular interest, given their frequency in JDM and their association with important disease features such as calcinosis, a major cause of morbidity. In addition to autoantibody status, age at disease onset has also been shown to influence the clinical phenotype and overall prognosis in JDM, although the nature of the relationship between age at disease onset and outcome has been variably reported [3, 4].

Here we analyse the clinical associations of anti-NXP2 measured by ELISA and protein immunoprecipitation within an extended and large cohort of juvenile myositis patients stratified by age of onset. We specifically assess the relationships between autoantibody status, calcinosis and disease activity.

## Methods 

### Patients

Patient serum samples and clinical data were obtained through the UK Juvenile Dermatomyositis Cohort and Biomarker Study (JDCBS). The JDCBS is a large cohort of UK patients with idiopathic inflammatory myopathies (IIMs), the majority with JDM [5]. Patients are recruited consecutively on presentation to paediatric rheumatology departments across the UK. Children and young people ≤16 years of age are included based on a diagnosis of definite or probable JDM or PM by Bohan and Peter criteria [6], as well as JDM or PM with overlap CTD features. Multicentre research ethics approval was obtained for the JDCBS and parental consent for children and consent or age-appropriate assent was obtained for all patients in accordance with the Declaration of Helsinki. The JDCBS Steering Committee approved this project and biological samples and clinical data were provided in accordance with JDCBS ethics and consent.

Remission in JDM was defined as a full-strength Childhood Myositis Assessment Score (CMAS) >48 [7], the absence of skin disease (no rash, no Gottron’s, no oedema and no ulceration) and a Physician’s Global Assessment Score (PGAS) <1. While this definition of remission has not been validated, all are standard outcome measures in JDM. Where possible, we also utilized the recently proposed PRINTO criteria for disease inactivity in JDM, defined as at least three of the following criteria: creatinine kinase ≤150, CMAS ≥48, manual muscle test (MMT) ≥78 and PGAS ≤0.2 [8].

### Autoantibody detection 

Immunoprecipitation of radio-labelled K562 cells was performed on all samples to determine the presence of autoantibodies. Anti-NXP2 ELISA was subsequently performed on all samples. Patients were classified as anti-NXP2 positive if a 140 kDa band was seen on immunoprecipitation and anti-NXP2 ELISA was subsequently positive.

#### Immunoprecipitation

Ten microlitres of sera were mixed with 2 mg of protein A–Sepharose beads (Sigma, St Louis, MO, USA) in immunoprecipitation (IPP) buffer (10 mM Tris–HCl, pH 8.0, 500 mM NaCl, 0.1% v/v Igepal) at room temperature for 30 min. Beads were washed in IPP buffer prior to the addition of 120 µl of [^35^S]methionine-labelled K562 cell extract in IPP buffer. Samples were mixed at 4°C for 2 h. Beads were washed in IPP buffer and Tris-buffered saline (TBS) (10 mM Tris–HCl, pH 7.4, 150 mM NaCl) before being resuspended in 50 µl of SDS sample buffer (Sigma). After heating, proteins were fractionated by 9% SDS-PAGE gels, enhanced, fixed and dried. Labelled proteins were analysed by autoradiography.

#### ELISA

ELISA was conducted as previously described [9], with some modifications. Ninety-six-well polystyrene plates were coated with rNXP2 (Origene Technologies, Rockville, MD, USA) at 4°C for 16 h. Samples were diluted to 1:200. Secondary antibodies were conjugated goat anti-human IgG/M (Sigma). Tetramethylbenzidine liquid substrate (Sigma) was then added. All samples were tested in duplicate and optical density was read using an automatic plate reader. The negative cut-off was defined as >3 s.d. above the mean of 42 normal healthy (adult) serum controls.

### Data analysis

Multivariate regression analyses were performed using generalized additive models within R [10], which allows for the possibility of non-linear relationships between covariates and response variables [11]. Within this framework, models were used to assess the significance of age at disease onset and anti-NXP2 in relation to calcinosis as well as disease outcome 2 and >4 years post-diagnosis. Disease duration was adjusted for. Potential differences between two groups were assessed using chi-squared analysis with Yates’s continuity correction. The Mann–Whitney *U* test was used to compare non-normally distributed continuous data.

## Results

Demographic data and key findings are summarized in Table 1.

**Table 1:** Demographic data and key findings of 285 patients analysed

	All patients	Anti-NXP2 positive
Female, *n*/*N* (%)	206/285 (72)	41/56 (73)
IIM subtype^a^, *n*/*N* (%)		
DM	242/285 (85)	55/56 (98)
PM	1/278 (0.4)	0
Overlap	33/285 (12)	1/56 (2)^b^
Age at onset, average (IQR), years	6.2 (4–10)	6.5 (4–10)
Time to diagnosis, average (IQR), months	4 (2–10)	4 (2–10)
Length of follow-up, average (IQR), years	9 (5–12)	9 (5–12)
Proportion with calcinosis, *n*/*N* (%)	94/283 (29)	24/56 (43), *P* = **0.025**
Lowest ever CMAS score, average (IQR)	40 (27–48)	29.6 (18–43), *P* < **0.001**

IQR: interquartile range; IIM: idiopathic inflammatory myopathy; CMAS: Childhood Myositis Assessment Score. ^a^In addition to the diagnostic categories listed, nine juvenile patients were labelled as other IIM or focal myositis. ^b^This patient had JDM–scleroderma overlap. Bold text indicates significant *P*-values. Demographic data were similar between anti-NXP2-positive and -negative groups.

### Autoantibody detection

Of the 365 patients enrolled in the JDCBS at the time of analysis, 285 (78%) had both data and serum available. This group was representative of the cohort as a whole, showing no significant differences in gender (72% *vs* 68% female), age at disease onset (median 6.2 *vs* 6.7 years) or JDM clinical type (12% *vs* 14% JDM overlap) compared with those patients not analysed.

Anti-NXP2 autoantibodies were identified in 56/285 (20%) patients. Two further patients were positive by anti-NXP2 ELISA but did not have a 140 kDa band on immunoprecipitation. Further analysis was carried out on only those patients definitively positive using both techniques.

### Calcinosis

**Fig. 1:**
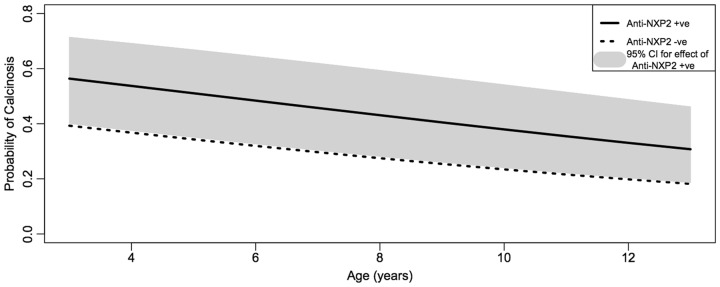
The effect of anti-NXP2 autoantibodies on the risk of calcinosis by age at disease onset (with 95% CI) A near-linear relationship is seen between younger age at disease onset and increased risk of calcinosis.

Overall, 33% of patients developed calcinosis during the follow-up period. Age at disease onset influenced the likelihood of calcinosis allowing for the duration of disease and there was a significant decrease in calcinosis with increasing age at disease onset [odds ratio (OR) 0.90, 95% CI 0.83, 0.97, *P* = 0.005]. Furthermore, this relationship with age proved to be near linear, a striking result given the flexibility of the model meant there were no prior constraints on its shape (Fig. 1).

There was a significant association with anti-NXP2 autoantibodies and calcinosis: 43% anti-NXP2-positive patients developed calcinosis *vs* 30% anti-NXP2-negative patients (OR 2.10, 95% CI 1.10, 4.01, *P* = 0.025 unadjusted for age, *P* = 0.039 adjusted). The presence of anti-NXP2 autoantibodies increased the risk of calcinosis after allowance for the possible effects of other variables, including age.

As some patients were recruited an appreciable time after their initial diagnosis, information on when calcinosis was first noted was not always available. However, seven children with anti-NXP2 autoantibodies developed calcinosis after their first study visit and all of these had anti-NXP2 autoantibodies present in blood samples predating calcinosis development (taken 2 months–4 years after disease onset). The average time to develop calcinosis in this group was 5.3 years (range 1.2–13 years). Thirty-two children without anti-NXP2 autoantibodies developed calcinosis after their first study visit and the average time to calcinosis was similar at 4.1 years after disease onset (range 0–10.9 years).

### Muscle weakness

Children with anti-NXP2 autoantibodies had a lower median lowest ever CMAS, corresponding to a greater degree of muscle weakness, compared with those without anti-NXP2 autoantibodies {29.6 [interquartile range (IQR) 18–43] *vs* 42 (IQR 31–48)} and this was statistically significant (*P* < 0.001). Furthermore, 53% of children with anti-NXP2 had a lowest ever CMAS ≤30, corresponding to a moderate or severe degree of weakness [7], compared with just 25% of those children without anti-NXP2 (*P* < 0.001).

### Disease outcome in JDM

Disease outcome was assessed 2 years post-diagnosis and at the last clinic visit, providing this was at least 4 years post-diagnosis (mean 7.9 years). Data at 2 years post-diagnosis were not yet available in children who had been diagnosed with JDM <2 years previously, had been recruited into the study >2 years post-diagnosis or had not been reviewed 20–28 months post-diagnosis. Information was available for 152 children 2 years post-diagnosis (25 with anti-NXP2 autoantibodies) and 136 children >4 years post-diagnosis (24 with anti-NXP2 autoantibodies).

Using our own predefined definition of remission, children with anti-NXP2 antibodies were less likely to be in remission 2 years post-diagnosis compared with those without anti- NXP2 (8% *vs* 32%; OR 0.23, 95% CI 0.05, 1.05, *P* = 0.04). Using the recently proposed PRINTO definition of disease inactivity in JDM [8], fewer children with anti-NXP2 had inactive disease 2 years post-diagnosis (37.5% *vs* 48%), but this did not reach significance. Outcomes >4 years post-diagnosis were similar in the two groups, irrespective of the definition used (our own criteria, 47% *vs* 50%; PRINTO criteria, 72% *vs* 73%).

## Discussion

This study shows that anti-NXP2 autoantibodies are the most common autoantibody identifiable in our JDM cohort and can be identified in one in five UK children with JDM. They are a potentially important clinical biomarker and can both facilitate diagnosis and provide important prognostic information, particularly with regard to calcinosis development.

Younger children with JDM are believed by some specialist clinicians to have more aggressive disease, but recent studies have challenged this view [4]. We have clearly demonstrated that children presenting at a younger age have a higher risk of developing calcinosis, a major cause of morbidity. The presence of anti-NXP2 autoantibodies substantially increases the risk of calcinosis across all ages.

Anti-NXP2 autoantibodies were associated with a greater degree of muscle weakness as determined by CMAS, suggesting more severe disease. This difference is of clinical significance and children with anti-NXP2 autoantibodies were more likely to have a moderate or severe degree of clinical weakness [7].

In JDM, the development of calcinosis has previously been associated with a delayed diagnosis, a chronic disease course and inadequately treated disease [12]. In our cohort, no difference in time from symptom onset to diagnosis was identified between the anti-NXP2-positive and anti-NXP2-negative groups. We investigated the prevalence of disease remission in JDM at two time points using two different definitions of disease inactivity. While a significant association between anti-NXP2 autoantibody status and more persistent disease activity 2 years post-diagnosis was suggested using our own definition of remission, the CIs were wide. We were unable to clearly demonstrate a significant relationship using the recently proposed PRINTO criteria, although the same trend existed. Persistent disease activity remains a possible mechanism driving calcinosis development in children with anti-NXP2 autoantibodies, but larger patient numbers and validated remission criteria are required to firmly establish the nature of any association.

In summary, anti-NXP2 autoantibodies are associated with features of severe disease in JDM. The development of calcinosis in JDM is influenced by both age at disease onset and anti-NXP2 autoantibody status and both factors must be considered when attempting to predict the risk of this clinically important complication.

**Rheumatology key messages**

- Earlier age at disease onset is associated with greater risk of calcinosis in JDM.
- Anti-NXP2 substantially increases the risk of calcinosis across all age groups in JDM.
- Anti-NXP2 autoantibodies are associated with features of severe disease in JDM.

## Supplementary Material

Supplementary Data
