# Supplementary material for: Calcinosis in juvenile dermatomyositis is influenced by both anti-NXP2 autoantibody status and age at disease onset
Source: Rheumatology (Oxford). 2014 Jul 1;53(12):2204–8. doi: 10.1093/rheumatology/keu259 (PMC4241891; doi:10.1093/rheumatology/keu259)
Supplement: Supplementary Data [file supp_53_12_2204__index.html]

Calcinosis in juvenile dermatomyositis is influenced by both anti-NXP2 autoantibody status and age at disease onset — Calcinosis in juvenile dermatomyositis is influenced by both anti-NXP2 autoantibody status and age at disease onset — Calcinosis in juvenile dermatomyositis is influenced by both anti-NXP2 autoantibody status and age at disease onset — Supplementary Data 

# Calcinosis in juvenile dermatomyositis is influenced by both anti-NXP2 autoantibody status and age at disease onset

## Supplementary Data

files

**Files in this Data Supplement:**

- Supplementary Data - docx file
