# Supplementary material for: Calcinosis in juvenile dermatomyositis is influenced by both anti-NXP2 autoantibody status and age at disease onset
Source: Rheumatology (Oxford). 2014 Jul 1;53(12):2204–8. doi: 10.1093/rheumatology/keu259 (PMC4241891; doi:10.1093/rheumatology/keu259)
Supplement: Supplementary Data [file supp_keu259_rhe-13-1497-File003.docx]

Supplementary material

The members of the Juvenile Dermatomyositis Research Group were as follows:

Dr Liza McCann, Mr Ian Roberts, Dr Eileen Baildam, Ms Louise Hanna and Ms Olivia Lloyd (The Royal Liverpool Children’s Hospital, Alder Hey, Liverpool), Dr Phil Riley and Ms Ann McGovern (Royal Manchester Children’s Hospital, Manchester), Dr Clive Ryder and Mrs. Janis Scott (Birmingham Children’s Hospital, Birmingham), Dr Sue Wyatt, Mrs Gillian Jackson, Dr Tania Amin, Dr Mark Wood and Vanessa VanRooyen (Leeds General Infirmary, Leeds), Dr Joyce Davidson, Dr Janet Gardner-Medwin, Dr Neil Martin, Ms Sue Ferguson and Ms Liz Waxman (The Royal Hospital for Sick Children, Yorkhill, Glasgow), Dr Mark Friswell, Professor Helen Foster, Mrs Alison Swift, Dr Sharmila Jandial, Ms Vicky Stevenson, Ms Debbie Wade, Dr Ethan Sen, Dr Eve Smith and Ms Lisa Qiao (Great North Children’s Hospital, Newcastle), Dr Helen Venning, Dr Rangaraj Satyapal, Mrs Elizabeth Stretton and Ms Mary Jordan (Queens Medical Centre, Nottingham), Dr Kate Armon, Mr Joe Ellis-Gage and Ms Holly Roper (Norfolk and Norwich University Hospitals), Professor Lucy Wedderburn, Dr Clarissa Pilkington, Dr N. Hasson, Mrs Sue Maillard, Ms Elizabeth Halkon, Ms Virginia Brown, Ms Audrey Juggins, Dr Sally Smith, Mrs Sian Lunt, Ms Elli Enayat, Mrs Hemlata Varsani, Miss Laura Beard, Miss Laura Kassoumeri, and Miss Katie Arnold (Great Ormond Street Hospital,  and UCL London), Dr Kevin Murray (Princess Margaret Hospital, Perth, Western Australia) Dr John Ioannou and Ms Linda Suffield (University College London Hospital).
